# Exploring the evolving concept of ‘patient ownership’ in the era of resident duty hour regulations—experience of residents and faculty in an internal medicine night float system

**DOI:** 10.1007/s40037-019-00540-9

**Published:** 2019-10-15

**Authors:** Vanessa Masson, Linda Snell, Diana Dolmans, Ning-Zi Sun

**Affiliations:** 1 University of British Columbia, Vancouver, British Columbia Canada; 2 McGill University, Montreal, Quebec Canada; 3 Maastricht University, Maastricht, The Netherlands

**Keywords:** Patient ownership, Professionalism, Duty hours, Night float

## Abstract

**Background:**

Despite the use of ‘patient ownership’ as an embodiment of professionalism and increasing concerns over its loss among trainees, how its development in residents has been affected by duty hour regulations has not been well described. In this qualitative study, we aim to outline the key features of patient ownership in internal medicine, factors enabling its development, and how these have been affected by the adoption of a night float system to comply with duty hour regulations.

**Methods:**

In this qualitative descriptive study, we interviewed 18 residents and 12 faculty internists at one university centre and conducted a thematic analysis of the data focused on the concept of patient ownership.

**Results:**

We identified three key features of patient ownership: personal concern for patients, professional capacity for autonomous decision-making, and knowledge of patients’ issues. Within the context of a night float system, factors that facilitate development of patient ownership include improved fitness for duty and more consistent interactions with patients/families resulting from working the same shift over consecutive days (or nights). Conversely, the increase in patient handovers, if done poorly, is a potential threat to patient ownership development. Trainees often struggle to develop ownership when autonomy is not supported with supervision and when role-modelling by faculty is lacking.

**Discussion:**

These features of patient ownership can be used to frame discussions when coaching trainees. Residency programs should be mindful of the downstream effects of shift-based scheduling. We propose strategies to optimize factors that enable trainee development of patient ownership.

**Electronic supplementary material:**

The online version of this article (10.1007/s40037-019-00540-9) contains supplementary material, which is available to authorized users.

## What this paper adds

While many are concerned about the potential detrimental effect of duty hour regulations on patient ownership, how the latter is affected by shift-based scheduling has not been well described. This qualitative study both adds to existing literature on the conceptual understanding of patient ownership and provides insights into the factors that enable its development in residents and how these were affected by the introduction of a night float scheduling system. Understanding the potential threats night float scheduling poses to patient ownership provides a starting point for development of system-level solutions.

## Introduction

The concept of *ownership*, or more specifically, *psychological ownership*, is well described within the organizational psychology literature [1]. When people develop a sense of ownership towards a target, they tend to seek to ‘protect and improve the [target] of the ownership’, which can be tangible (e.g. an object) or intangible (e.g. a process) [1]. In medicine, ownership of patient care, often referred to as *patient ownership*, is widely recognized as a key element of medical professionalism and as a critical skill to develop during residency training [2]. It has traditionally been described as ‘the philosophy that one knows everything about one’s patients and does everything for them’ [3], and as ‘being assigned the care of a patient 24 h a day, 7 days a week; being responsible for the patient’s management and eventual disposition; and being the one person in charge of decision-making’ [4]. Since the advent of resident duty hour regulations, multiple authors have raised concerns about their impact on the development of patient ownership, fearing that shift-based scheduling might threaten acquisition of this important skill [3, 5–8]. The applicability of existing conceptual definitions of patient ownership [2, 4] in the context of newer scheduling systems that are compliant with duty hour regulations is unclear. Furthermore, how residents’ development of patient ownership may be influenced by duty hour regulations, such as through the use of night float scheduling, remains unknown. In this study, we sought to explore how internal medicine residents and faculty describe resident patient ownership in the context of an in-patient teaching unit with duty hour regulations, and to understand how its development in residents might have been affected by the introduction of shift-based scheduling.

## Methods

### Context and participants

In 2010, the McGill University internal medicine residency program (Montreal, Canada) implemented a 12-hour shift-based night float system on six medical clinical teaching units distributed across its three teaching hospitals. This scheduling system consists of two daytime resident teams at each hospital, each covering one of the two clinical teaching units and one two-resident night-time team covering both teaching units. The day teams comprise a fixed set of residents for 4 weeks, and the night team residents work overnight shifts for 1–2 consecutive weeks and are relieved of all daytime clinical and academic activities. A detailed description of the study setting, including the night float system, was previously published [9] (See Appendix 1 in the online Supplementary Electronic Material).

Nine clinical teaching unit attending physicians, all 3 hospital-specific internal medicine residency program directors, and 18 internal medicine residents participated in this qualitative study. In order to allow for maximal phenomenal variation [10], the participants were recruited equally from each of the three teaching hospitals and half of the resident participants had previously worked within the traditional 24-hour scheduling system. This study was conducted in accordance with the ethical standards of the McGill Faculty of Medicine Institutional Review Board (IRB) and with the 1964 Helsinki Declaration and its later amendments. The initial study conducted in 2013 received full IRB approval (IRB study number A01-E11-13B). The current study, which involved analysis of previously collected and de-identified data, received IRB exemption. Individuals were invited by email and informed consent was obtained in person from all individual participants included in the study. No incentive was provided for participation.

### Data collection and instrument

Data were collected via 30 semi-structured in-depth interviews in 2013. All interviews were conducted by N.‑Z. S, who was a full-time master’s student in Health Professions Education at the time of data collection. The interviewer, N.‑Z. S, was not involved in clinical supervision at the time of data collection and, as such, had no power differential with any of the study participants. Participants were asked to comment on attributes of medical professionalism within the context of the new night float scheduling. Those with experiences with the previous 24-hour scheduling system were asked to contrast their experiences prior to and following the implementation of the new night float system. The interview protocol was previously published [9] (see Appendix 2 of the Electronic Supplementary Material).

### Data analysis

The interview transcripts were previously analyzed by Sun et al. with the aim of understanding the impact of duty hour regulations on the workplace and professionalism in general [9]. It became apparent early on during the initial data collection/analysis that patient ownership was a complex theme that sparked rich discussions during the interviews. However, this could not be examined in depth during the initial data analysis given the latter’s broader scope. We therefore performed a more focused analysis of the raw data from all 30 interview transcripts with the specific aim of exploring how patient ownership is conceptualized by our participants and how its development in residents might have been affected by shift-based scheduling as a result of duty hour regulations. We chose to use qualitative description methodology, as described by Sandelowski [10], which is ideally suited to produce an accurate accounting of the subject under study in everyday language. We coded the interview transcripts in an inductive and iterative manner using qualitative content analysis [10]. Given the lack of a universally accepted conceptual definition for patient ownership, we used a conventional approach to content analysis and generated themes inductively based on interview data without imposing preconceived categories [11]. Entire transcripts were first reviewed to get a sense of the whole. Statements where ‘patient ownership’ (or a variant thereof) was spontaneously mentioned were then repeatedly reviewed to achieve immersion. From this, we coded statements that directly referred to patient ownership and subsequently expanded our coding by letting previously emerged themes iteratively inform our analysis until no new theme or subtheme emerged. The themes were subsequently compiled, and a framework was built based on their interrelationships. All data were independently coded by two investigators (V. M. and N.‑Z. S.). Coding alternated between faculty and resident transcripts to allow for emergence of any thematic divergence between the two groups. The results were compared and discrepancies between the two investigators’ coding were resolved through discussion until consensus was reached. All authors participated in the development of the conceptual framework. We did not encounter new themes in our analysis beyond the 10th interview transcript. Nonetheless, all 30 interview transcripts were analyzed to assess how strongly other participants identified with the emerging themes. All data related to patient ownership were coded in all transcripts.

## Results

**Fig. 1:**
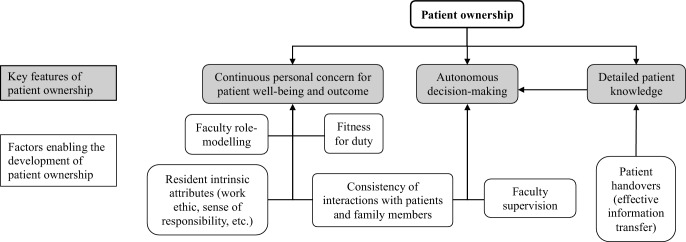
Key features of patient ownership and factors that enable its development in residents

Following our primary analysis, we generated a conceptual framework, depicting resident patient ownership as described by both faculty members and residents, and some of the factors affecting its development in the setting of night float scheduling (Fig. 1). Below, we present the themes and a figure illustrating the links between them. Supporting participant quotes use [S] for ‘staff’ physician and [R] for ‘resident’. Findings were very similar amongst residents and faculty with no significantly divergent views identified.

### Key features of resident patient ownership in the context of night float scheduling

Participants identified three elements as key features of patient ownership: continuous personal concern for patient well-being and outcome, professional capacity for autonomous decision-making, and possessing detailed patient knowledge.

First and foremost, both residents and faculty deemed that displaying *continuous personal concern for their patients* that transcends the limits of scheduled shifts or duty hours was a core feature of patient ownership.

*Even if we are home and somebody is […] taking care of our patient for a few hours, we still have ownership of that patient. We still care about what happens to patients [… so] we end up calling the person at night and making sure that things have been done […] to make sure that we are taking care of the patient. [R01]*

Second, participants agreed that having sufficient professional capacity for decision-making was an essential part of patient ownership.

*[…] patient ownership [… means] that [you are] the primary person responsible for them; [… that] even if you call in a consult[ant] to help you with one of the issues, that it’s you who has to take what the consultant suggested or thought and […] integrate that with all the other issues […] that’s your responsibility. [R18]*

Third, stakeholders perceived that holding *detailed patient knowledge* was a fundamental aspect of patient ownership. They defined this as having a global understanding of the details relevant to their patients, rather than any individual piece of information.

*[… patient ownership is] knowing the patient, who they are, where they come from, what their main medical issues are, what they might need […] I’m talking about the bio-psycho-social, all the medical issues, as well as all the potential family issues [… and] psych issues […]—everything. [R10]*

### Factors enabling the development of resident patient ownership in the context of night float scheduling

First, participants highlighted the importance of *faculty role modelling* in helping residents develop *continuous personal concern* for their patients, one of the key elements of patient ownership. Working with attending physicians who embodied this concept facilitated their own ability to develop such sense of responsibility. It was felt that night float-based scheduling constituted a challenge for trainees, given the lack of faculty presence at night.

*[…] on the night float service, […] you’re operating in a very isolated fashion […] whereas during the day you have this opportunity to model more experienced senior [clinicans who can] guide [trainees] along and show them ways to react. [R03]*

Second, study participants agreed that *residents’ intrinsic attributes*, such as their personal work ethic and sense of responsibility, played a major contribution in their ability to develop *personal concern*, and consequently, ownership of patients under their care, especially in settings with scarce or absent faculty presence, as discussed above.

*[…] There are some people who will arrive to do the night float as their penitence. They just need to get it over with and keep the patients alive and do those admissions, but they really don’t want to do the admissions and they’re counting the days until the end. […] And, then there are those who really are interested and are learning from the patients. […] So patient ownership is really a characteristic of individual residents and not necessarily of the duty hour system. [S02]*

Third, stakeholders described observing that the limited work hours enhanced residents’ *fitness for duty*, which enabled them to feel more engaged in their interactions with patients and their family members and, thus, more easily develop *personal concern* for their patients’ well-being and outcome. Study participants who had experience with the previous 24-hour scheduling system highlighted that feeling more rested with the night float system made it easier for them to develop patient ownership.

*I think during the day, because you’re less tired and more present, you have more time to sit down and speak with patients, speak with families, work on these very humane aspects of being a professional […] [R03]*

Fourth, the *consistency of interactions with patients and their family members *was also deemed to greatly influence residents’ ability to develop *personal concern* for their patients and their care. Within the night float system, trainees worked a series of consecutive days or nights, which allowed for more consistent resident-patient contact and positively influenced trainees’ development of patient ownership. Those with experience with the prior 24-hour call scheduling system observed a marked improvement in consistency of resident-patient contact with the night float system, which in turn improved patient ownership. This was attributed to the removal of the post-call days that were inherent to the previous 24-hour call system and the associated interruptions in individual resident’s contact with their assigned patients.

*When it comes to patients, the more you interact with [them], the more you […] start feeling like they are your responsibility. We all go through that, where there are certain people that you follow, and then you worry about them and then […] you’re not on [duty] anymore but you are [… still] checking to see what is happening. [S04]*

Additionally, the *consistency of interactions with patients and their family members* was also felt to enhance trainees’ professional capacity for autonomous decision-making by allowing them to develop a more thorough understanding of their patients’ medical and social situations.

*[Because] you are the same night float team for the entire week or entire 2‑weeks, […] you know the patient well, […] you know the family, [… so] you’re much more inclined to do discussion of goals of care and procedures on the spot because you feel comfortable as opposed to delaying them to the next morning. [R06]*

Fifth, stakeholders identified that *supervision of residents by faculty members *played a crucial role in their *ability to autonomously make patient care decisions. *Residents feel a stronger sense of patient ownership when they are entrusted to make decisions related to patient care. With the night float system, stakeholders felt that limited contact between the night float residents and the clinical teaching unit attending, who is primarily present during daytime, made it difficult for the night float residents to develop a sense of where a patient’s care is headed. This posed the risk of limiting these residents’ ability to make decisions and inhibiting development of patient ownership.

*[When a patient is admitted] at night time, the day team still takes the ownership over it. Because the overall hierarchy is there and the leadership is there to tell you where you’re headed. On the other hand, somebody gets admitted on the day time, that sense of ownership […] doesn’t necessarily get translated to the night […] because the day team is the one who is going to make all the decisions anyway, so why bother doing this. [S03]*

Finally, *effective information transfer* was identified as essential in enabling residents to develop *detailed patient knowledge* and patient ownership. With an increased number of handovers in a shift-based scheduling system, high quality, detailed patient handover was felt to be essential for patient ownership.

*I think it comes down to the quality of sign-outs. […] if the day team is not giving an adequate sign-out, not telling them enough about issues, not calling in to talk to the senior resident overnight to tell them everything that’s going on, I think it’s then detrimental to the person who is on nights because they don’t know or understand the big issues that are going on. [R16]*

## Discussion

In this study, staff physicians and residents identified three key features of patient ownership within the context of an internal medicine in-patient service with duty hour regulation: continuous personal concern for patients, professional capacity for autonomous decision-making, and having detailed knowledge of patients’ issues. Previous studies have also highlighted autonomy, commitment, and knowledge as essential elements of patient ownership within the specific context of psychiatry and internal medicine residency programs [2, 4]. This echoes our findings, supporting the importance of these consistent key elements to the concept of patient ownership across not only different subspecialties, but also different scheduling systems.

From a theoretical perspective, our participants’ description of patient ownership strongly parallels the concept of psychological ownership. Pierce et al. describe three mechanisms by which people come to feel psychological ownership towards a target: by ‘investing self into the target’, by ‘controlling the target’, and by ‘coming to intimately know the target’ [12]. If patient care is considered the target of ownership, parallels can be drawn as follows: continuous personal concern for patients (investing self into patient care), professional capacity for autonomous decision-making (controlling patient care), and detailed knowledge of patients’ issues (coming to intimately know the details of patient care).

Some authors have expressed concerns over the implied notions of power and dominance in the word ‘ownership’, which can potentially undermine collaborative care and patient empowerment [13]. The appropriateness of the word ‘ownership’ is further challenged by the study by Lingard et al., which outlines the collaborative complexity that can arise from involvement of different medical teams in patient care and the lack of ‘stable locus of control or authority’ in such context [14]. However, despite the potential paternalistic and individualistic connotations of the theme ‘professional capacity for decision-making’, we believe that, taken as a whole, the three key features of patient ownership we identified support a patient ownership construct that is in keeping with the predominant discourse in the literature, which is patient-centred with notions of commitment, responsibility, accountability, advocacy, and continuity [3, 15].

In our study, we did not find significant divergence between resident and faculty perceptions of patient ownership or of the factors enabling its development in residents, which contrasts others’ observations [2, 4]. This difference may be explained by the fact that, while others have explored how residents and faculty define patient ownership as constructed by each group’s own experience, we asked our faculty participants to specifically reflect on resident patient ownership, which may not be the same as their conception of faculty patient ownership.

Analyzing the impact of night float scheduling on resident patient ownership, we identified a number of both positive and negative noteworthy effects. Comparing night float with the previous 24-hour scheduling system, study participants felt that being more rested and having a more consistent interaction with patients over consecutive days without the interruptions caused by post-call days made it easier for residents to develop patient ownership. This finding of more consistent patient interaction echoes those of Mathew et al., who also observed that night float scheduling positively affected senior residents’ patient ownership on internal medicine clinical teaching units as a result of improved continuity of care during daytime hours [16]. This contrasts with other authors’ portrayal of the negative impact of duty hour regulations on continuity of patient care and patient ownership in surgical residents [17–21], which raises interesting questions about the influence of specialty-specific context and culture.

The increased number of handovers was felt to have the potential to limit trainees’ knowledge of patient issues and consequently their sense of patient ownership. Additionally, residents and staff physicians both agreed that the limited presence of supervising clinicians at night creates a more permissive work environment due to reduced role modelling and guidance for decision-making and may impede the development of patient ownership in residents with weaker work ethics. The importance of resident autonomy for the development of patient ownership has been highlighted by several other studies [8, 15, 22]. Our study identifies the additional nuanced view that, when left without adequate supervision at night, trainees sometimes feel uncomfortable making autonomous decisions, which can deleteriously impact ownership taking. This phenomenon has previously been described by Olmos-Vega et al., who highlighted that residents retreat to being passive observers when they perceive a lack of autonomy within an unsafe learning environment, such as when the supervisor is unavailable [23]. In our study, this translated into the risk of compromising residents’ development of patient ownership.

Those with experience with the prior 24-hour call scheduling system highlighted the positive impact of the marked improvement in consistency of resident-patient contact, both during daytime and night-time. We believe that the structure of our night float system, which employs a stable team of residents independent from the daytime teams (thus protecting the latter’s integrity) over a number of consecutive night shifts (thus providing consistency in patient contact), played an important role in fostering patient ownership.

Deconstructing the concept of patient ownership helps us to better understand its key features, which can help inform assessment criteria and guide learning environment optimization to promote its development. Clearly articulated assessment criteria can inform selection of entrustable professional activities and associated milestones as we transition to competency-based medical education. The key descriptors of patient ownership that we found help to complement the developing literature [2, 4] in providing a better understanding of this concept and how it relates to the broader concept of psychological ownership.

We identified a few elements of night float scheduling that may impair development of patient ownership in residents and argue that these can be mitigated by institutions and training programs. For instance, residency training programs might consider facilitating faculty contact with residents working night shifts, including encouraging open discussions between residents and faculty regarding expectations related to when residents should engage in autonomous decision-making at night (with review of decisions delayed until the next morning) and when they should seek immediate guidance. Curricula should aim to integrate opportunities to support residents’ autonomous decision-making. For example, using simulations to practice challenging clinical scenarios might empower trainees to feel more capable of making decisions when left unsupervised. Our findings suggest that consistency of resident-patient contact is a key element of ownership. Night float systems should therefore be purposefully designed to provide stability of both daytime and night-time teams and thus permit consistent resident-patient interaction for the entire duration of residents’ clinical teaching unit (and night float) rotations. Finally, given the important impact of information transfer on patient ownership, we suggest that residency programs should work towards adopting the principles of continuity-enhanced handovers as proposed by Arora et al. and encouraging a culture where both the leaving and receiving parties are held accountable for providing high-quality handovers [24].

Our study has a number of limitations. This was a single-centre study focusing on data gathered from one residency training program within the specific context of internal medicine clinical teaching units. The specific night float scheduling system used at our institution might also differ in part from others used elsewhere [25]. However, we hope the detailed description of our study context (Appendix 1 of the Electronic Supplementary Material) will help inform the transferability of our findings.

## Conclusion

In conclusion, our study provides insights into the concept of patient ownership and how it may have been affected by duty hour regulations. The themes we identified can be used as a framework to guide discussions when providing feedback to trainees. Residency programs should aim to mitigate the downstream effect of shift-based scheduling by implementing system-level changes and educational opportunities, as well as promoting entrustment of trainees’ decision-making. Future research should be aimed at exploring how patient ownership is acquired by trainees and expressed in different specialty and clinical settings, at understanding how it is enacted and perceived in the context of interprofessional (as opposed to inter-physician) teamwork, and at assessing interventions designed to foster its development in trainees.

## Caption Electronic Supplementary Material

Study context (appendix 1) and interview protocol (appendix 2)
