# Supplementary material for: Exploring the evolving concept of ‘patient ownership’ in the era of resident duty hour regulations—experience of residents and faculty in an internal medicine night float system
Source: Perspect Med Educ. 2019 Oct 15;8(6):353–9. doi: 10.1007/s40037-019-00540-9 (PMC6904378; doi:10.1007/s40037-019-00540-9)
Supplement: Supplementary file 1 — Study context (appendix 1) and interview protocol (appendix 2) [file 40037_2019_540_MOESM1_ESM.docx]

Appendix 1: Study context

The internal medicine residency program at McGill University comprises of approximately 120 medical resident physicians yearly, whose training levels range from postgraduate year (PGY) 1 to 3. They are distributed across three different teaching hospitals located in Montreal, Canada. Our dataset was collected as part of a previous study, which focused on the impact of duty hour restrictions on the workplace and their effect on medical professionalism in general (9).

Our dataset consists of 30 semi-structured interviews conducted by NZ.S. in mid-2013 following implementation of a 12-hour shift-based night float system by the internal medicine program. This night float system was implemented in two phases between July 2009 and July 2010, on the medical clinical teaching units across all three training hospitals (two units per hospital). Its implementation was voluntary and in anticipation of upcoming provincial duty hour regulations, which restricted maximum shift length to 16 consecutive hours starting July 2012. More detail about the actual implementation process can be found elsewhere (25).

In this model, the night float team, which comprises of 1 junior (PGY-1) and 1 senior (PGY-2 or 3) resident, is assigned to a series of 5 consecutive nights, from 8PM to 8AM, for a total duration of 1 or 2 consecutive weeks. When on duty, the night float team covers in-patient care on the medical clinical teaching units, handles acute issues on other inpatient non-teaching medical units, and serves as the primary resuscitation team across the hospital. During the daytime, from 8AM to 5-6 PM, teams comprising of four residents (two PGY-1s, one PGY-2, one PGY-3) and two medical students takes over clinical care on the clinical teaching units (one team per unit). The night float team is released from both daytime academic and clinical duties. During the evening, between 5-6 PM and 8PM, one “late” resident (either a junior or a senior) from each day team stays on his/her respective unit to handle acute issues after receiving handover from their teammates. They are responsible for handover to the night float team at 8PM. Within this system, patient handovers occur on 3 different occasions over any 24h period: from the night float team to the day team, from the day team to the late resident, and from the late resident to the night float team. To facilitate handover of patient information, all team members utilize typed, standardized patient lists, which summarize key information such as past medical history, reason for admission, active issues, medications, allergies, and medical care plan.

Appendix 2: Interview Protocol

As previously published (Sun NZ, Gan R, Snell L, Dolmans D. Use of a Night Float System to Comply with Resident Duty Hours Restrictions: Perceptions of Workplace Changes and Their Effects on Professionalism. Academic Medicine. 2016;91(3):401-8.)

1. Could you briefly tell me about your role and the extent of your experience on medical CTUs?

Use following probing questions as necessary:

i). (for attending physicians only) How many years of experience have you had working on medical CTUs with residents?

ii). (for attending physicians only) Were you trained at McGill University for your core internal medicine residency?

iii). (for residents only) How many medical CTU rotations have you had so far? 1b. Could you tell me briefly why you are interested in participating in this interview?

2. What is your understanding of medical professionalism in general and in the context of medical CTUs?

Use following probing questions as necessary:

i). In your opinion, what attributes define medical professionalism?

ii). Among these, if you can only choose one, which one is the most important in your opinion?

iii). In your opinion, which attributes of medical professionalism are particularly relevant to the work of residents on medical CTUs during daytime? At night time?

3. Reflecting specifically on medical professionalism and residents’ work (substitute “residents’ work” with “the work of your peers” if the interviewee is a resident) on medical CTUs under the night-float system, please tell me about...

3a. positive experiences you have had with respect to residents’ work (substitute “residents’ work” with “the work of your peers” if the interviewee is a resident) during daytime.

3b. positive experiences you have had with respect to residents’ work (substitute “residents’ work” with “the work of your peers” if the interviewee is a resident) at night time.

3c. disappointments you have had with respect to residents’ work (substitute “residents’ work” with “the work of your peers” if the interviewee is a resident) during daytime.

3d. disappointments you have had with respect to residents’ work (substitute “residents’ work” with “the work of your peers” if the interviewee is a resident) at night time.

4a. How do the experiences you just mentioned compare to your experience with residents’ work (substitute “residents’ work” with “the work of your peers” if the interviewee is a resident) on medical CTUs under the traditional 24-h call system?

4b. Overall, would you say that the night-float system had a positive or negative impact on medical professionalism on medical CTUs? (Invite the participant to elaborate)

4c. In your opinion, which professional attributes have been most affected (positively or negatively) by the transition to the night-float system? (Invite the participant to elaborate)

5a. If you are in charge, and can make one single change, with the exception of altering the present duty hour system, to promote medical professionalism on medical CTUs, what would you change? (Invite the participant to elaborate)

5b. (ONLY if time allows) If you can make two other changes, what would they be? And why?

6. (only for current or past program directors)

6a. During which academic years were you appointed as core or site program director?

6b. Thinking specifically about CTU rotations before and after implementation of the NF system, have you had experiences with respect to the concept of professionalism in your capacity as core/site program director in addition to experiences you had as a CTU attending physician? If yes, could you elaborate?

7. Invite the participant to think about the following statements, elaborate on their reactions, and explore rationale and beliefs behind these.

In the literature, duty hour restriction has been found to affect the following professionalism issues either positively or negatively. Please compare each statement below with your own beliefs and discuss with me your thoughts on the subject. Please note that the wording of the statements is purposefully NOT consistently reflective of current literature.

- Duty hour restriction has a positive impact on residents’ altruism.
- Duty hour restriction has a positive impact on residents’ patient ownership.
- Duty hour restriction leads to better teamwork.
- Duty hour restriction necessitates better teamwork.
- Duty hour restriction has a positive impact on residents’ empathy towards patients.
- Duty hour restriction has a positive impact on residents’ competence in providing medical care.
- Duty hour restriction reduces medical errors by residents.
- Duty hour restriction puts residents’ self-interest in conflict with patients’ interest.
- Duty hour restriction leads to shift worker mentality.

(ONLY if time allows)

8a. (for attending physicians only) In addition to working on CTUs, do you play other roles in your institution?

8b. (if answered yes to 8a) Any comments about the impact of duty hour restrictions on professionalism from these other perspectives?
